# A Biomimetic Visual Sensing Framework: Unsupervised Orientation Topographic Mapping via Self-Organizing Neural Networks

**DOI:** 10.3390/biomimetics11060435

**Published:** 2026-06-18

**Authors:** Tianqi Chen, Zhiyu Qiu, Yuki Todo, Zheng Tang

**Affiliations:** 1 Graduate School of Information Science and Technology, The University of Osaka, Suita 565-0871, Osaka, Japan; ch1n.weather@osaka-u.ac.jp; 2 Division of Electrical Engineering and Computer Science, Kanazawa University, Kanazawa 920-1192, Ishikawa, Japan; qiuzy1916@stu.kanazawa-u.ac.jp; 3 Faculty of Electrical, Information and Communication Engineering, Kanazawa University, Kanazawa 920-1192, Ishikawa, Japan; 4 Institute of AI for Industries, Chinese Academy of Sciences Nanjing, Nanjing 211135, China; tang@iaii.ac.cn

**Keywords:** artificial visual system, machine learning, unsupervised learning, bio-inspired model, orientation detection, image classification, noise robustness

## Abstract

In this study, we propose a biologically inspired Self-Organizing Map-based Artificial Visual System (SOM-AVS) for unsupervised orientation detection in static images. By combining a biologically motivated front-end visual processing module with an unsupervised SOM layer, the proposed system captures key characteristics of early-stage visual processing, including localized orientation-sensitive responses and structured feature organization. The model enables the structure of distinct orientation-related representations without requiring labeled data, forming organized response patterns across the neural map. Experimental results demonstrate robustness under various conditions, including noise corruption, restricted perceptual experience, and limited training samples. Furthermore, the model shows adaptive behavior when exposed to new stimuli after initial training, indicating its potential to reflect experience-dependent adjustments in representation. These findings suggest that SOM-AVS provides a useful framework for exploring self-organization mechanisms in artificial visual systems and for developing biologically inspired perception models.

## 1. Introduction

The visual system is a highly efficient, multi-channel processing mechanism that forms the foundation for human information perception and behavior regulation, and it is specially optimized for this purpose [1,2,3]. Studies have shown that over 80% of the information in mammals’ brains is derived from visual input, while the retina plays a crucial role in the early preprocessing of visual signals [4,5]. These signals are from rods and cones in the photoreceptor cells, which convert light into neural activity [6]. Among them, cone cells are responsible for color vision and divide the input into three channels based on wavelength of the light [7,8]. In addition, the retina processes visual signals in its inner nuclear layer, including bipolar and horizontal cells, which integrate and modulate the input signals and pass the information to the lateral geniculate nucleus (LGN) and higher cortical regions [9,10,11].

Within the broad spectrum of visual functions, orientation detection is a fundamental and essential ability. Orientation detection supports the detailed analysis of spatial arrangements in static scenes, which underlies object recognition and scene understanding [12,13]. This functional structure has strong neural foundations and is deeply embedded in the visual system’s architecture through coordinated processing pathways that involve hierarchical computations spanning retinal circuits to cortical areas [14,15,16,17,18].

Orientation detection is primarily supported by neurons in the primary visual cortex (V1), which are organized into columnar structures that exhibit orientation selectivity [19]. These columns show continuous angle preferences across the cortex, creating a topographic map of visual edges [20]. However, orientation selectivity is not a fixed feature; research has shown that this property is highly plastic and dependent on visual experience during development [21,22,23,24]. For example, animals without exposure to certain orientations exhibit weaker neural responses to those orientations [23]. This reveals that cortical organization depends on stimulus-driven self-organization.

Biological plasticity enables efficient neural representations through optical stimuli via unsupervised learning processes. This sharply contrasts with traditional machine learning approaches. Supervised methods require large labeled datasets and extensive computational resources, which is markedly different from biological brain mechanisms [25,26]. This aligns with the idea that general representations can be learned through unsupervised pretraining. Later, these representations can be aligned to specific labels via fine-tuning with a small amount of annotated data. This process is similar to how infants learn to map sensory experiences to semantic labels. They do so through limited social interaction [27,28].

It is a crucial consensus in visual neuroscience that the biological visual system utilizes unsupervised processes wherein orientation selectivity emerges natively through experience-dependent self-organization. In other words, the mammalian visual system leverages unsupervised pathways that self-organize through repeated, passive exposure to environmental optical stimuli [29,30,31,32]. This biological paradigm closely demonstrates the same working flow with the modern engineering convention that generalized multi-modal representations can be established via unsupervised pre-training and subsequently aligned to semantic labels through minimal annotated fine-tuning [27,33]. Furthermore, the continuous mathematical optimizations and backpropagation loops driving modern deep learning models have further emphasized the fundamental divergence between bio-inspired self-organizing mechanisms and conventional artificial networks [34,35].

Bio-inspired models, however, currently suffer from significant limitations. Many rely on fixed filters or handcrafted rules, limiting their adaptability [36]. A problem that cannot be ignored is that, while orientation detection has been modeled in single- and multi-channel settings, no unified framework exists that can handle both conditions simultaneously without resorting to artificial solutions, such as empty-channel padding. Many architectures rely too heavily on fixed convolutional templates or handcrafted sensory rules, thereby severely curtailing their environmental adaptability. A critical and long-ignored problem in this domain is that, while orientation detection has been modeled in either isolated single-channel or multi-channel settings, no unified framework exists that can handle both conditions simultaneously without resorting to artificial, non-biological solutions such as empty-channel padding [36]. This operational discrepancy poses a serious challenge for conventional deep networks: models optimized for monochromatic features fail to scale to multi-channel chromatic (RGB) visual streams without executing structural alterations or parameter resetting. Consequently, the inability to adaptively unify single- and multi-channel streams within a common topology remains a fundamental gap for current computational frameworks.

Our previous research developed retinal-inspired orientation detection models, but these primarily focused on local orientation selectivity at the cellular level. Studies suggest that learning shapes how local orientational signals are integrated into global perception, yet the precise plasticity mechanisms underlying this transition remain unclear within computational frameworks, constituting a fundamental gap.

As a result, the use of unsupervised processes in the biological visual system is now viewed as a promising strategy for developing biologically plausible image-processing systems. For instance, bio-inspired models such as spiking neural networks (SNNs) can develop direction sensitivity from raw event-based inputs. More studies have shown that motion discrimination improves through unsupervised stimulus exposure [37]. These principles inspire the design of unsupervised computational vision models. However, these models tend to work on the dynamic image instead of the static image, showing the limitations of SNNs.

Orientation detection, particularly the classification aspect, is closely related to unsupervised image classification tasks. In traditional approaches, images are encoded into a latent space and reconstructed via decoders; then, the representations are clustered using methods like K-means. More advanced methods, such as DeepCluster, employ end-to-end learning to improve classification accuracy [33,38]. Compared with handcrafted clustering, these methods benefit from the richer features learned through autoencoder-based networks. Present unsupervised classification models include IIC (Invariant Information Clustering), and SCAN (Semantic Clustering by Adopting Nearest Neighbors) [33]. Despite their effectiveness, these models are often criticized for their lack of biological interpretability, known as black-box models.

However, existing computational models either rely on handcrafted orientation encoding or lack mechanisms for topological reorganization and plasticity. In 1982, Teuvo Kohonen proposed the Self-Organizing Map (SOM), an unsupervised neural network that maps high-dimensional input data onto a low-dimensional grid while preserving topological relationships. This fulfills the needs of biologically inspired modeling [39,40]. SOMs reconstruct feature maps and categorize not only motion directions but also orientations based on biologically plausible rules [41]. These findings highlight the potential of SOMs to simulate specific functions of the visual system.

In this study, we investigate the capacity of SOMs to perform orientation detection in an unsupervised setting. We propose a biologically motivated input encoding scheme based on the activation patterns of retinal-inspired local orientation-selective neurons, which extracts spatial information from image sequences and feeds it to the SOM. Through experiments involving target removal and network reconstruction, we simulate use-dependent plasticity observed in biology and demonstrate the self-organizing and adaptive nature of the model. Results show that the SOM is capable of distinguishing orientation information, and in certain cases, is even capable of re-learning or enhancing features post-modification. These findings suggest that SOM-based systems offer a promising direction for biologically inspired vision models with unsupervised learning capabilities.

In summary, orientation detection represents a cornerstone function of the biological visual system. This study focuses on investigating how SOM can support activity-dependent topological reorganization. It relies on stimuli-driven learning and structural self-organization, both in functional objectives and anatomical substrates. These characteristics not only highlight the adaptive essence of vision but also provide guiding principles for designing artificial vision systems that are more faithful to biological principles and capable of unsupervised learning.

## 2. Methods

### 2.1. Bio-Inspired Artificial Visual System

**Figure 1:**
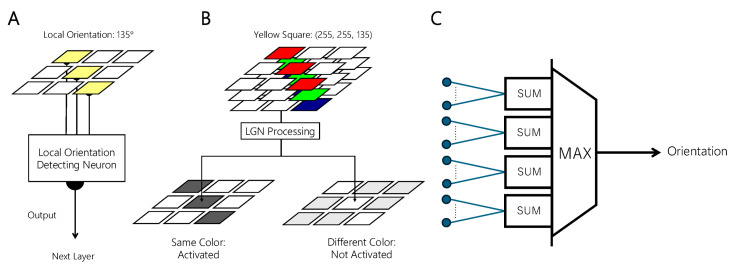
Structure of orientation detection: (**A**) local orientation detection using AVS neuron models; (**B**) LGN-filtered activation across three color channels; (**C**) population-based integration in the global detection layer.

Based on the foundational work of Hubel and Wiesel on the visual system and the detailed cellular architecture they uncovered, we previously developed a series of progressively refined bio-inspired artificial visual system (AVS) models. These models simulate the response mechanisms of neurons in the biological visual system and offer a strong biological explanation. The overall structure of the AVS system is shown in Figure 1, covering the basic structure of orientation detection.

The AVS models are designed to perform the essential visual function: the detection of four specific orientations in static objects, which is regarded as fundamental to the biological visual system. The architecture of our AVS consists of two primary components: a local detection layer responsible for classifying orientations at the pixel level, and a global detection layer that integrates these local signals to determine the overall classification. This biological structure is inspired by the theory of simple cells and complex cells, along with the roles of inner nuclear layers in early visual processing. As shown in Figure 1A,B, the local detection layer can adopt different neuron models, including the dendritic neuron models for orientation detection and the Hubel–Wiesel model-inspired orientation detection AVS models, which are mainly used in this study.

Input images are first processed by the photoreceptor cells, which mimic the first stage of the visual pathway. In this step, rod and cone cells convert light stimuli into electrical signals, depending on light intensity and wavelength, via different channels. The signals are then transmitted to the local detection layer. The number of nodes in this layer corresponds to the resolution of the input image in pixels, and each node is associated with a number of types of neurons, each specialized in detecting a specific classification. These neurons generate pixel-level feature maps that act as classification filters.

Although different AVS models may use varying mechanisms, their pixel-level responses tend to be similar when applied to the same dataset.

The AVS model adopted in this paper is implemented as a robust, noise-resistant orientation detection block, based on the structure proposed in a hybrid model [42]. As illustrated in their work, this AVS block mimics a three-stage biological vision process involving the retina, lateral geniculate nucleus (LGN), and primary visual cortex (V1). The retina stage computes local color contrasts using a dynamic threshold within a 3×3 receptive field. The LGN layer applies a center-surround suppression filter to eliminate background redundancy and enhance local contrast. Specifically, for a 3×3 receptive field patch *p*, the central pixel is regarded as $p_{1,1}$, we first compute a local threshold θ(p) based on pixel differences:

(1) $$\theta \left(p\right)=\sum_{i=0}^{2}\sum_{j=0}^{2}\sqrt{\frac{{\left(p_{i,j}-p_{1,1}\right)}^{2}}{8}}+3,$$

where the constant threshold offset (+3) serves as a bio-inspired baseline regulatory gain that is structurally coupled with the spatial side-dimension ($\sqrt{9}=3$) of the localized 3×3 receptive field. This parameter operates as a mathematical stabilizer within the early visual circuit, preventing the dynamic scaling threshold θ(p) from prematurely collapsing to zero when processing the same patches characterized by negligible local pixel variance, thereby sustaining the framework’s baseline noise-filtering efficacy. Only when a neighboring pixel value $p_{i,j}$ falls within the range $p_{1,1}-\theta <p_{i,j}<p_{1,1}+\theta$, it is considered similar to the center and retained; otherwise, it is suppressed. This logic ensures that only spatially consistent local structures are preserved, enhancing orientation-selective responses and reducing noise-induced activations.

The V1 layer consists of fixed convolutional templates corresponding to four orientations ($0^{\circ }$, $45^{\circ }$, $90^{\circ }$, $135^{\circ }$), allowing the model to simulate the orientation selectivity of simple cells, as visualized in Figure 1A. Complex cells then integrate these outputs to select the most responsive orientation per location.

In this model, each orientation corresponds to one color-coded output channel. As shown in Figure 1B, neurons activated by LGN processing are highlighted in dark gray, while non-activated neurons remain in light gray. Each of the three color channels in the input is processed independently to account for different wavelength sensitivities, shown as separate pathways originating from the yellow square.

This biologically grounded block offers both spatially localized orientation detection and robustness to visual noise, and has demonstrated its effectiveness in various visual conditions.

Similarly, for global orientation detection, the model evaluates the activation statistics of local orientation neurons and assigns the final label based on the dominant response across the visual field [43,44]. As shown in Figure 1C, local neurons with the same orientation preference project to a shared global orientation neuron, whose activity reflects the population-level consensus. This approach simulates the distributed and hierarchical computation patterns observed in the visual cortex.

Despite their strong biological basis, traditional AVS models share a key limitation: they rely on predefined classification rules or supervised label settings. The output orientation categories are either explicitly encoded in the architecture or fixed by the designer, leaving little room for adaptive adjustment. As such, these models do not exhibit the experience-dependent plasticity observed in natural visual systems.

To address this gap, in our current framework, we retain the AVS as a front-end feature extractor, but connect it with a SOM to enable unsupervised learning of orientation representations. The AVS module transforms raw image inputs into spatially structured, orientation-selective activation patterns. These outputs serve as biologically meaningful and compact representations, making them well-suited as input to the SOM model. This integration combines the interpretability of biologically inspired preprocessing with the flexibility and adaptability of unsupervised learning—laying the foundation for self-organized orientation perception.

### 2.2. SOM-Based AVS

In the first part of our Section 2, we present a concise overview of our previously developed supervised AVS models, highlighting their underlying biological principles and strong robustness. These models provide the conceptual foundation for introducing the unsupervised model, SOM. The AVS framework also serves as a basis for generating biologically meaningful inputs to the SOM. Notably, the AVS module used prior to SOM can be highly customized. Depending on the task, the AVS can be flexibly adjusted by selecting different neuron models, filter settings, and activation thresholds (e.g., noise filter in the present research or 4-class orientation classifier in this research). The AVS is not trained using labels but operates as a fixed, biologically inspired preprocessing module.

The SOM, introduced by Teuvo Kohonen in the early 1980s, is an unsupervised neural network algorithm designed to produce a low-dimensional, structured representation of high-dimensional input data while preserving topological relationships [39]. This process closely resembles the self-organizing properties of the biological brain, particularly in sensory processing regions such as the visual cortex.

In the visual system, neurons in V1 exhibit topographically organized receptive fields, meaning that nearby neurons respond to similar visual stimuli, a phenomenon observed in retinotopic maps [18,20]. Similarly, SOMs create structured maps in which neighboring neurons represent similar input features. This alignment between biological self-organization and SOM learning has led researchers to propose SOMs as computational models for feature representation in the brain [45,46].

**Figure 2:**
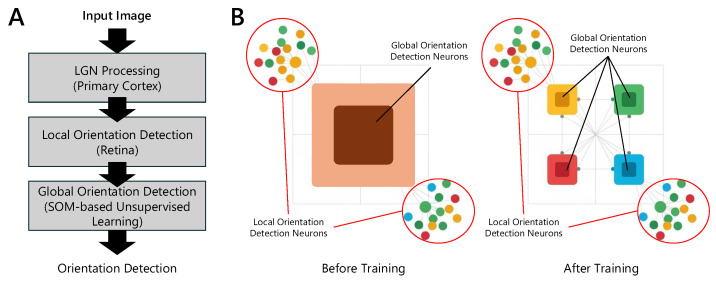
Structure and training effect of SOM-based AVS: (**A**) model architecture combining local orientation detection and SOM-based global detection layers; (**B**) visualization of connection patterns before and after training.

In this study, we combined the AVS for 4-class orientation detection with SOM to construct a SOM-based AVS that enables the formation of a global detection system through repeated exposure to stimuli, providing a more natural method for classification in AVS models. Figure 2A illustrates the overall framework of the model. The general structure of this model is developed upon previous fixed AVS architectures, yet it introduces a major methodological shift. Traditional AVS systems rely on pre-defined global decision maps or supervised tags, which prevent experience-dependent plasticity [36]. In contrast, the current SOM-AVS architecture replaces static decision rules by combining the local detection layer in a bio-inspired structure with an adaptive, self-organizing matrix layer. The primary methodological difference lies in transforming local, cell-level receptive responses into non-overlapping spatial-indexed vectors that allow the global SOM layer to execute experience-dependent representation adjustments naturally without external oversight.

The local orientation detection layer retains the same mechanism as it does in the AVS block, with a modified output format. In previous research on the orientation classifier, the identity of each local orientation detecting neuron was unimportant; we only needed to focus on binary values indicating whether the neurons were activated. In SOM-based AVS, we use a 4-dimensional vector (1,O) as the output for each local detecting neuron, where *O* = 4 for this orientation detection task. The output indicates the identity of each activated neuron. Each dimension corresponds to a specific orientation class. For this orientation detection task, the four dimensions typically correspond to horizontal, vertical, and two diagonals. When a neuron is activated, it outputs a matrix where the dimension corresponding to the detected orientation contains the index of the neuron’s position, while all other positions remain zero. If a neuron is not activated, it does not produce any output. The index *I* is calculated using the following formula:

(2) $$I=\frac{a+(h+S_{1}\cdot w)}{S_{2}}$$

In the formula, to prevent the output from being entirely zero, we introduce a constant offset *a*. In this study, we set a=1024, the middle value of the unfolded pixel length. Specifically, because the inputs are configured as 32×32 images spanning exactly 1024 spatial grid coordinates, choosing a=1024 mathematically enforces that the resulting positional postcode index *I* maintains unique, non-overlapping, non-zero boundaries across all pixel locations. Furthermore, the scaling coefficient $S_{2}$ is symmetrically fixed at 1024 to normalize the coordinate index magnitudes back to a bounded scale, effectively mitigating gradient or scale disparities against the randomly pre-seeded weight matrices during Euclidean similarity comparisons. The variables *h* and *w* represent the neuron’s horizontal and vertical coordinates, respectively. This encoding does not explicitly impose spatial structure, but provides a compact representation that allows SOM to capture spatial correlations implicitly. Since we use 32 × 32 images as input, the maximum values of *h* and *w* are 31. The coefficient $S_{1}$ ensures that *h* and *w* are mapped uniquely to *I*. We set $S_{1}=32$. The coefficient $S_{2}$ is a scaling factor that prevents excessively large output values; in this study, we set $S_{2}=1024$.

Next, we pass these indexed data into the SOM-based global detection layer. In this layer, we apply the SOM principle to cluster neurons corresponding to each orientation class. Clusters are defined based on the spatial grouping of BMUs corresponding to similar input patterns [39]. According to the SOM mechanism, when an input is received, the Best-Matching Unit (BMU) must be determined, whether during the training or detection process. The BMU is the neuron in the SOM layer whose weight vector is most similar to the input vector. The similarity is measured using Euclidean distance, which quantifies the difference between the input vector *I* and all weight vectors $w_{i}$. This process corresponds to a winner-take-all mechanism, analogous to lateral inhibition observed in biological neural circuits [18,20,39]. The BMU is computed as follows:

(3) $$\mathrm{BMU}=\arg\min_{i}∥I-w_{i}∥$$

During the training process, once the BMU is identified, the weights of neighboring neurons are updated, with the magnitude of change decreasing as the distance from the BMU increases. The weight update process in the SOM model is governed by the following equations:

- Weight Update Equation:
  Here, $w_{new}$ represents the updated weight, while $w_{old}$ is the previous weight. The learning rate η controls the step size of the update. The neighborhood function Λ determines the influence of the BMU on its surrounding neurons. The term $\left(I\;-\;w_{old}\right)$ represents the difference between the input vector and the current weight.
  (4) $$w_{new}=w_{old}+\eta \cdot \Lambda \cdot \left(I\;-\;w_{old}\right)$$
- Neighborhood Function:
  The neighborhood function Λ defines how much a neuron’s weight is influenced based on its distance *d* from the BMU, where *d* is measured using Euclidean distance. The influence decreases exponentially as the distance increases, following a Gaussian distribution. The standard deviation σ controls the spread of this function.
  (5) $$\Lambda =e^{-\frac{d}{2\sigma^{2}}}$$
- Standard Deviation of the Neighborhood Function:
  The parameter σ is proportional to the map size *M* and decreases over time as the learning rate η decreases. The map size *M* refers to the width of the SOM weight matrix. In this study, we set *M* = 96, meaning the SOM weight matrix has a size of 96 × 96. This function ensures that in the early stages of training, a larger neighborhood of neurons is influenced, while in later stages, only neurons closer to the BMU are updated.
  (6) $$\sigma =\frac{M\cdot \eta }{2}$$
- Learning Rate Decay:
  The learning rate η decreases linearly over time, where *t* is the current epoch, and *T* is the total number of training epochs This gradual decay allows for significant weight adjustments in the early training phase and fine-tuned adjustments in later stages, stabilizing the learned representations. This design is inspired by the critical period observed in biological systems, in which synaptic plasticity in the visual cortex is most active during a postnatal “critical period” and diminishes thereafter, rather than being a direct model of biological plasticity [22,47]. Therefore, in our model, both the learning rate and the neighborhood radius progressively decrease as training advances, mirroring the biological decline in plasticity over time.
  (7) $$\eta =1-\frac{t}{T}$$

Figure 2B illustrates the schematic diagram of the connection between local and global detecting neurons before and after training. The objects at the top represent the local orientation detection layer, while those at the bottom represent the global orientation detection layer. The lines in the middle indicate the connections between the two layers. Each node in the local layer contains multiple neurons detecting different orientations. In the initial state, the connections between two layers are disorganized. After training, neurons detecting the same class form clustered connections to specific regions in the global orientation detection layer. Ultimately, we can determine the global orientation by analyzing the number of activated neurons within each region.

## 3. Result

### 3.1. Dataset Statement and Preprocessing

In this section, we present several groups of experiments to demonstrate the superior performance of our proposed SOMAVS in orientation detection tasks. All modeling experiments and comparative baselines were executed within a Python 3.10 environment utilizing the PyTorch 2.1 framework. Hardware operations were standardized on a workstation equipped with an NVIDIA RTX 3090 GPU (8 GB VRAM). Under this specification, the baseline training footprint for the SOM-AVS architecture averaged approximately 4.4 min per 100 epochs. To ensure complete scientific reproducibility, the source package and the scripts for generating the synthetic dataset have been made accessible under reasonable terms at the data availability section. The dataset used in our experiments consists of over 50,000 synthetic images, each of size 32×32 pixels with three color channels in a certain regulation, which are red, green, and blue. To simulate different lighting and background conditions, we modified the three-channel values of each pixel across the dataset. Additionally, the dataset contains objects of five different sizes—12, 16, 32, 64, and up to 128 pixels—with each size represented by more than 10,000 samples, ensuring objects have different locations and shapes. These objects were programmatically generated with sizes ranging from 12 pixels (larger than a single receptive field of the AVS model to ensure biological plausibility) to 256 pixels (one-fourth of the image size), which allows for effective recognition while minimizing irrelevant background noise.

The image dataset consists of two parts: binary images and full-color images. The binary images are single-channel, where 0 represents the background, and 1 denotes the object. This set of images is used to evaluate model performance in a basic scenario, where image complexity is intentionally limited. In contrast, the full-color images encompass nearly all possible variations, with both objects and backgrounds randomly colored. In some cases, one may be nearly random while the other remains a solid color. This set of images introduces more complex real-world conditions, allowing for a robustness test of the model’s performance. Crucially, within the context of biological vision simulation, establishing a viable alternative to this programmatically regulated dataset remains exceptionally challenging. Unlike standard computer vision engineering benchmark suites designed for high-level downstream semantic tasks, our framework operates strictly from a computational neuroscience perspective [26,48]. To rigorously observe and verify the microscopic self-organization of cortical orientation columns, isolating low-level geometric primitives from confounding high-level semantic contexts is a well-established methodological necessity in developmental neurobiology [22,24]. Standard natural image repositories contain unconstrained, complex contextual dependencies that render controlled environmental variables’ isolation mathematically intractable for evaluating low-level feature wiring. Recent computational paradigms have confirmed that unsupervised learning on structured, low-level sensory primitives is sufficient and necessary to generate efficient, topographically accurate neural representation geometries without complex semantic overhead [29,31]. Consequently, relative to the targeted topological mapping mechanism under evaluation, conventional mainstream networks engineered for global abstract semantics exhibit specific representation limits when orientation constraints are omitted, suggesting that this programmatically regulated dataset formulation provides an effective, controlled environment for analyzing bio-inspired perceptual self-organization.

Each object was assigned one of four orientations: $0^{\circ }$, $45^{\circ }$, $90^{\circ }$, or $135^{\circ }$. These angles were chosen because they can be effectively distinguished by a 3×3 receptive field, which is the typical size used by AVS to simulate local orientation-selective processing that enables reliable recognition while avoiding useless information. Considering the square shape of image pixels and the properties of simple and complex cells in the retina and LGN, these four angles represent the most distinguishable orientations. Indeed, the human visual system cannot perceive infinitely fine angular differences; instead, it categorizes orientations based on perceptual thresholds. In digital images, orientation perception is derived from the distribution of RGB values across local regions. In addition, it is important to note that even with very large object sizes, reliable orientation detection is not always guaranteed. In fact, due to the windowing effect, a convolutional kernel may only observe a flat or ambiguous portion of the object (e.g., a uniform stripe ), which lacks clear orientation features. This demonstrates that both too small and too large object sizes can degrade orientation cues, further complicating learning in standard CNNs that rely on limited receptive fields.

To prepare input for the SOM, each image was first processed by the AVS system. For each pixel, an activation value of 0 or 1 calculated by AVS was assigned to indicate whether a neuron tuned to a particular orientation was activated. This transformed the input from shape (c,h,w) to (h,w,O), where *O* is the number of orientation classes (in this case, the value of *O* is 4). The resulting orientation maps were then reshaped into a 2D matrix of size (h×w,O) for input into the SOM. To retain spatial information, we generated a location encoding postcode ranging from 0 to h×w−1 and multiplied it element-wise with the reshaped activation map. To avoid large value disparities across channels, we added a constant (1024) to all non-zero entries. Next, the orientation maps were separated into different channels, and the value of the corresponding channel was moved to the aimed orientation angle. For example, a vector $\left(V_{1},V_{2},V_{3},V_{4}\right)$, the multi-channel encoding becomes something like $\left(\left(V_{1},0,0,0\right),\left(0,V_{2},0,0\right),\left(0,0,V_{3},0\right),\left(0,0,0,V_{4}\right)\right)$. After flattening, the final input to the SOM is a matrix of size (h×w×O,O), which is (4096,4) in our dataset configuration.

### 3.2. Pre-Experiment

To benchmark the effectiveness of SOMAVS, we conducted several pre-experiments using other unsupervised orientation learning methods.

First, we applied IIC and contemporary contrastive self-supervised paradigms (e.g., SimCLR) to establish baseline benchmarks for unsupervised visual representation learning [25,33,49]. However, the pre-experimental results were highly unsatisfactory, revealing a systemic limitation of mainstream networks when applied to downstream absolute orientation detection tasks. Contemporary self-supervised frameworks rely fundamentally on aggressive spatial data augmentations to force the network to learn semantic invariances by using mechanisms such as random flipping and isotropic rotations [33]. In the strict context of absolute orientation detection, however, such transformations inherently violate geometric consistency by altering the very directional features under evaluation (e.g., inadvertently transforming a $0^{\circ }$ primitive into an oblique vector). Consequently, when these orientation-disrupting augmentations are omitted to preserve absolute angle consistency, these models suffer from representation bottlenecks and alignment difficulties during unsupervised clustering. Under these restricted training conditions, only random cropping remains as a viable augmentation candidate. However, the synthesized object sizes in our dataset only span up to one-fourth of the entire visual field, the probability of capturing robust, object-relevant orientational features during random cropping is exceptionally low, or even nothing exists in the cropped area. Without sufficient foreground guidance, the underlying CNN architecture inevitably tends to optimize invariant features from homogeneous background regions, which are completely devoid of orientation information. Conversely, even when the target object is sufficiently large, the cropping window occasionally extracts only a textureless, uniform, or highly ambiguous local sub-patch, which similarly lacks global orientation features. In both scenarios, the representation spaces learned as invariant features by conventional mainstream pipelines predominantly originate from these non-informative, redundant regions. This yields misleading clusters and suboptimal feature mapping, encounter severe optimization constraints in mirroring raw input, the topology-driven SOM-AVS framework.

In addition, we tested a wide range of autoencoder and K-means pipelines. We experimented with both simple and complex architectures for feature extraction. To guarantee full evaluation fairness, all comparative deep baselines were rigorously optimized. The models were trained using the AdamW optimizer with a baseline learning rate of $\eta =1\times 10^{-4}$ and a weight decay coefficient of $1\times 10^{-4}$. The learning rate decay was managed by a StepLR schedule rather than a continuous curve. Crucially, the training duration was fixed at 200 epochs based on our 1000-epoch preliminary experiments; these pre-experiments revealed that CNN architectures typically plateaued after 200 epochs, following a clear overfitting trajectory in which test set accuracy initially rose and subsequently declined. The latent feature dimensions were universally constrained to 1024, the same as the SOM-AVS identical to the dimensions configured for the SOM-AVS framework, before being projected onto standard *K*-means clustering layers where the cluster number was structurally fixed at k=4 to align with the orientation categories. To ensure absolute fairness in the evaluation protocol and structural reproducibility, model selection for all baseline deep architectures was standardized by extracting latent features immediately prior to the final global pooling layers. The evaluation protocol strictly utilized the same frozen feature representations across all iterations, projecting them onto identical *K*-means layers where clustering accuracy was calculated based on the Hungarian algorithm mapping matrix, ensuring a direct and uncompromised performance comparison. Standard spatial data augmentations, such as random flipping and isotropic rotations, were intentionally deactivated to prevent the destruction of directional semantics, leaving stochastic cropping as the primary pipeline constraint. Specifically, we tried CNNs with 4 convolutional layers, shallow CNNs with single-layer encoders, LeNet-based architectures, ResNet family (from ResNet-18 to ResNet-101), EfficientNet-B0, and Vision Transformers (ViT). We even implemented hybrid CNN-ViT combinations that incorporate transformer blocks after convolutional stages. Despite this diversity in model design, the final clustering results remained poor across all configurations. We observed that learned latent spaces failed to reflect orientation-related differences, and the clusters formed by K-means lacked semantic consistency. This further emphasizes the difficulty of learning biologically relevant orientation features via conventional unsupervised approaches. This outcome implies that while modern deep architectures yield limited improvements in classification tasks when augmented with a topological component, their inherent global pooling mechanisms still pose substantial limitations for converging on micro-level orientation tasks without dedicated structural constraints.

These observations motivated us to explore a biologically inspired alternative. By leveraging orientation-selective pre-processing through AVS and the topology-preserving unsupervised learning capability of SOM, our proposed SOMAVS model avoids the shortcomings of the models.

### 3.3. Basic Training Results

**Figure 3:**
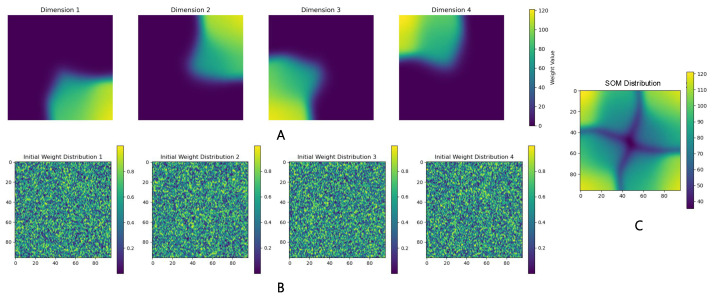
(**A**) Initial weights sampled from the SOM before training. (**B**) Weight distribution after training. (**C**) Combined classification boundaries visualized via thresholding.

Following the flow above, we did some experiments. Specifically, we trained the model for 100 epochs. Figure 3 illustrates the weight distribution before and after training. The model exhibits natural self-organization: distinct regions emerge in the SOM weight map, each corresponding to one of the classification categories. Initially, the weights are randomly set by the program in Figure 3A. After training, we identify the regions associated with each classification based on the weight distribution. As shown in Figure 3B, each classification occupies a distinct area in the SOM space, where only certain weight dimensions remain active. When all dimensions are combined, these active regions tile the entire map without overlap, regardless of the number of categories.

In Figure 3A, four distinct regions emerge for four orientations. Each region represents the receptive field of neurons selective for a specific category, and the category can be determined by the nonzero dimensions of the weights. Since the region boundaries are often smooth and gradual, we introduced a threshold α to accurately define the boundaries of each region. Neurons with weights exceeding this threshold in a particular dimension are assigned to the corresponding category. Given that the minimum input value is approximately 1.0, we set α=0.5 in this study. Rather than an arbitrary heuristic, this selection acts as a strict mathematical median threshold within the normalized neural activation bounds (0.0 to 1.0). By adopting the exact numerical midpoint, it objectively bifurcates active representation zones from silent background coordinates without introducing subjective tuning bias. To better illustrate the classification boundaries, merge all weight maps into a single representation in Figure 3C. Figure 3C shows the boundary structure of the organized map for four orientations.

**Figure 4:**
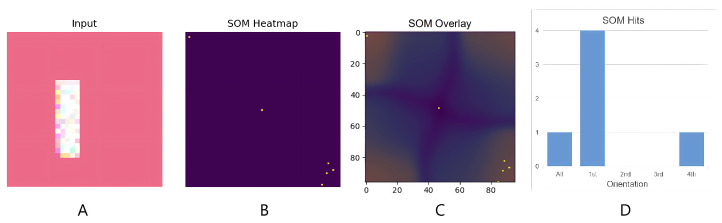
(**A**): Image of the vertical-oriented object. (**B**): The distribution map of activated neurons on the global orientation detecting layer. (**C**): Overlay image of activation map and merged SOM. (**D**): The BMU matching counts of the input image.

After training, the SOM self-organized into a complete orientation map, with distinct regions dedicated to each of the four orientations. To verify whether the model has been successfully trained, we randomly generated a sample image containing a vertical 80-pixel object. The sample image was input into the SOM-AVS model, where the local orientation layer first processed it and activated multiple orientation-selective local neurons. These activations were subsequently transmitted to the SOM-based global orientation detecting layer, which in turn activated the most responsive global neurons, producing a topographic activation pattern as illustrated in Figure 4. As shown in Figure 4A, the input stimulus contains a clearly defined vertically oriented 80-pixel-size object. The resulting global activation map is shown in Figure 4B, and the overlay with the classification boundaries from the saved weight map is shown in Figure 4C. The specific distribution of BMU activations across the network is captured in Figure 4D. Notably, orientation indices 2 and 3 register zero SOM hits under this vertical test trial. This absence is not an algorithmic artifact or failure; rather, it provides strong empirical confirmation of the network’s high tuning selectivity. Given that the input stimulus is perfectly aligned with the vertical axis ($90^{\circ }$), neurons representing orthogonal or oblique preferences are successfully silenced via the simulated lateral inhibition mechanism surround suppression behaviors, which is a direct computational parallel to V1 center. In order to isolate these discriminative mappings, the central area of the activation scale (all the orientational neurons are activated), which demonstrates the position of high-frequency non-specific background in the image, was normalized. The highly localized concentration of activations within the designated overlay coordinates rigorously demonstrates that the unsupervised SOM-AVS framework effectively partitions and encodes visual boundaries without requiring external labels.

The detection results of SOMAVS on binary and full-color (3-channel) images are presented in Table 1. To ensure the reliability of the orientation detection and rule out coincidence, we repeated the experiments five times with different random seeds. The values in the table represent the mean accuracy ± standard deviation.

The accuracy difference between binary and full-color images suggests that SOMAVS maintains robust performance across input modalities, although the accuracy on full-color images is slightly lower. Upon further inspection, we found that this drop is largely attributable to orientation ambiguities in objects with nearly equal height and width. In such cases, the model occasionally classifies an object’s orientation by 90 degrees into another label, since the spatial features along orthogonal axes become indistinguishable. Nonetheless, this issue has limited practical impact, as the affected samples account for a small fraction of the dataset and the model still consistently organizes orientation-specific responses in a topologically meaningful way.

### 3.4. Few-Shot Learning

**Figure 5:**
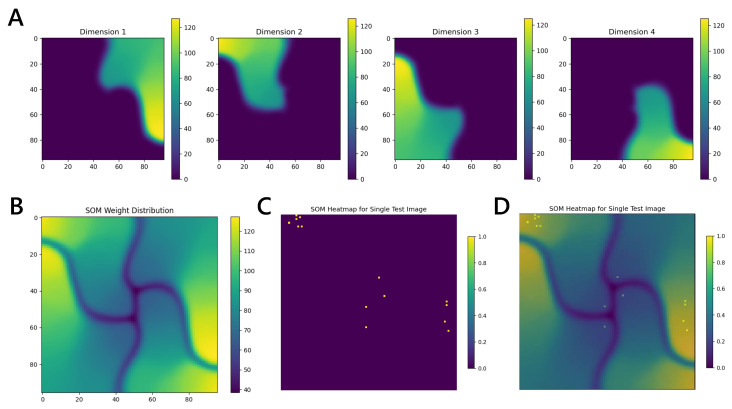
Few-shot orientation detection. (**A**) Trained weights for four orientation classes. (**B**) Merged visualization. (**C**) Activation map for test orientation. (**D**) Overlay of weight and activation map.

To further evaluate the data efficiency and robustness of our model, we trained it using only 50% or 10% of the complete training dataset. Despite the significantly reduced data, the SOM-AVS model retained its ability to self-organize into functionally meaningful maps. The experimental results, after training, showed that the model still successfully separated the four orientation classes into distinct regions. As shown in Figure 5A, the trained weight map reflects clear clustering of neurons for each orientation class, though the distribution appears slightly more distorted, and the position of each region has changed compared to full-data training. In Figure 5B, we merge all four dimensions into a single 2D view for easier visualization, which still reveals organized regional separation. The activation map from test samples (Figure 5C) confirms that neurons are appropriately activated according to their preferred orientations. Furthermore, we overlaid the trained weight map onto the activation distribution in Figure 5D to show spatial correspondence between weight tuning and neural response. These results in Table 2 indicate that despite reduced training data, the model maintains orientation selectivity and spatial consistency in its activations.

Notably, even under the few-shot condition, the principal activation zones remained nearly unchanged compared to the full-data version, demonstrating stable feature representation. This result suggests that the SOM-based global detection mechanism not only generalizes well from limited data but also exhibits spatial consistency in its activations. This variation stems from the nature of the SOM learning process: when the first input is presented, its BMU is determined by randomly initialized weights, and the neighborhood around the BMU begins to adapt. Since the first training input is randomly drawn, the final location of each orientation in the region is effectively randomized between runs. Nevertheless, the distinction between classes remains intact, and the model’s performance is not compromised. The activation map generated from test data still correctly activates the neurons associated with the input orientation, confirming that accurate classification is preserved even under low-data conditions.

### 3.5. Ablation Analysis

To further evaluate the effectiveness of our learned SOM module, we conducted a series of ablation experiments by integrating the SOM block into several representative neural network architectures. These experiments were designed to assess whether the inclusion of the SOM contributes to improved classification performance, especially under conditions where conventional CNN-based models may struggle, such as the influence of our dataset on traditional unsupervised neural network models.

Specifically, the dataset consists of images with relatively small spatial dimensions and high semantic density per pixel due to high object-to-background contrast, especially of all the local orientation features in pixels. These properties make it difficult for models relying on global feature aggregation to learn effective representations, as the spatial compression may result in the loss of critical orientation-related details, which are necessary in CNN and ViT models.

Nonetheless, by incorporating the trained SOM module as a component block, we observe a consistent improvement in performance. The SOM effectively guides the feature space toward more meaningful cluster boundaries, since it has learned to organize and separate orientation features in an unsupervised manner. This alignment appears to benefit large-scale architectures by providing localized orientation priors, thus allowing them to make more informed predictions even without access to explicit labels during training.

In our ablation study, we tested multiple ResNet variants (ResNet-18, 34, 50, and 101), conducting five independent trials per configuration. Given the relatively poor performance across these models, we collectively refer to them as ResNet-style CNN. Similarly, we tested EfficientNet-B0 through B7 and aggregated the results under the label EfN-style CNN. Additionally, we evaluated a hybrid model that combines a ViT encoder with CNN components, referred to as ViT-hybrid-style CNN. Finally, we included the original AVS model without the SOM module as a baseline, although AVS was not originally designed for clustering or unsupervised tasks.

Table 3 summarizes the accuracy of each model category with and without the SOM block. As a result, the inclusion of the SOM consistently leads to an increase in classification accuracy across all model families, indicating its general utility as a biologically inspired pre-processing and feature alignment module for unsupervised learning tasks. We also implemented Normalized Mutual Information (NMI) as an additional evaluation metric to assess the clustering quality of each model, particularly for unsupervised settings where label alignment is nontrivial. The observed trends in NMI were consistent with the accuracy results, further validating the effectiveness of SOM-based feature structuring. For brevity, “Acc”, “w/” and “w/o” are used throughout the tables and figures to denote “Accuracy”, “with” and “without,” respectively.

The experimental result shows that the inclusion of the SOM consistently leads to an increase in classification accuracy across all styles of models. The result indicates SOM’s general utility as a biologically inspired pre-processing and feature alignment module for unsupervised learning tasks. We also assessed the clustering quality of each model; the observed trends in NMI were consistent with the accuracy results, further validating the effectiveness of SOM-based feature structuring.

### 3.6. Restricted Development Simulation

**Figure 6:**
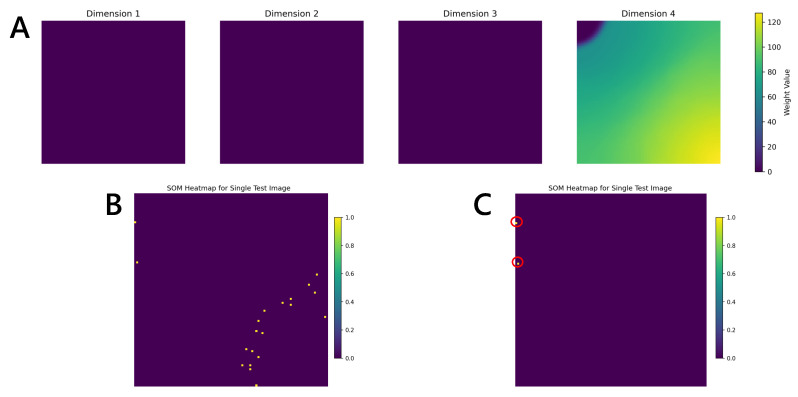
(**A**) Weight for each orientation after training. (**B**) The distribution map of activated neurons for trained orientation on the global detecting layer. (**C**) The distribution map of activated neurons for other orientations. The shot points are highlighted by red circles.

To further investigate whether the SOM-AVS model is consistent with biological developmental processes, we conducted control experiments simulating restricted perceptual experiences. As shown in Figure 6, we removed data of several orientations from the training set to simulate individuals that develop in special environments.

In the first condition, the model was trained using data corresponding to only a single orientation, while keeping all other experimental parameters (e.g., learning rate, total number of epochs) identical to the baseline setting. The resulting weight maps (Figure 6A) showed that only the dimension corresponding to the trained orientation retained nonzero weights across the global orientation detecting layer. In contrast, the weights associated with the untrained orientations were suppressed to zero. We then analyzed the activations of the global orientation detecting layer when test data from both trained and untrained orientations were input. As shown in Figure 6B, inputs corresponding to the trained orientation activated a broad region of the global layer. Conversely, inputs from untrained orientations resulted in sparse, highly localized activation (Figure 6C), which we have highlighted with red circles. This behavior reflects the self-organizing nature of the SOM, where only the trained orientation establishes a dominant representation, while untrained orientations fail to form substantial receptive regions. This result aligns with the findings of Blakemore et al [24]. In their experiments on cats, exposure to a specialized environment significantly increased the number of neurons in the visual cortex responsive to the experienced orientation, while reducing the number of neurons sensitive to other orientations. A similar effect was observed in the orientation detection task, as the experimental results showed.

**Figure 7:**
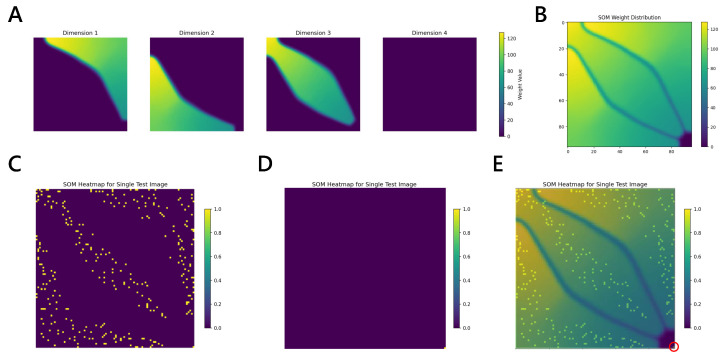
Orientation detection with 3-orientation training. (**A**) Weight map. (**B**) Merged view. (**C**) Activation for trained orientations. (**D**) Activation for untrained orientations. (**E**) Overlay showing mapping mismatch for untrained data (red circles).

We further conducted additional experiments using datasets containing subsets of orientations to simulate varying degrees of developmental restriction. The results consistently mirrored those observed in the triple-orientation and double-orientation training scenario. As shown in Figure 7A, when trained on a 3-orientation dataset, the weight map of the global orientation detecting layer only exhibited organized regions for the trained orientations. For clearer visualization, the merged representation across four dimensions is presented in Figure 7B. The corresponding activation map for test stimuli from the trained orientations (Figure 7C) shows the activation position similar to all orientations experiments, and localized within the relevant regions. In contrast, activations for untrained orientations are scattered and weak, appearing only in the gaps between dominant areas (Figure 7D). To highlight this contrast, Figure 7E overlays Figure 7B–D, with red circles indicating untrained orientation mappings that fail to occupy the clear zones. This pattern remained consistent even when the number of available orientations was further reduced.

**Figure 8:**
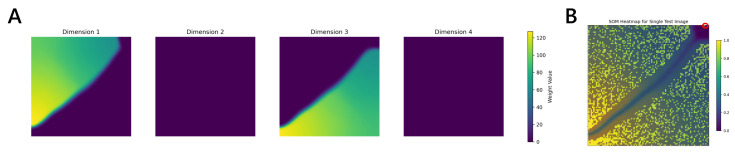
Orientation detection with 2-orientation training. (**A**) Weight map. (**B**) Mapping positions, red circles highlight untrained responses.

As shown in Figure 8A, the global orientation-selective weights were developed only for experienced orientations in the double-orientation training scenario. Figure 8B visualize the mapping relationship between weight distribution and neuron activation. The experimental results illustrate the absence of well-formed receptive fields for untrained orientations (highlighted by red circles).

These phenomena align with the classic work by Blakemore, which showed that early visual deprivation leads to selective responsiveness in the visual cortex [24]. The similarity reinforces the biological plausibility of our model’s developmental mechanisms. Together, these results suggest that our SOM-based model captures the fundamental developmental principle that perceptual maps form through selective exposure. The consistency across orientation, which is one of the most fundamental functions in visual system, further supports the robustness and generality of this bio-inspired mechanism.

### 3.7. Plasticity After Training

To evaluate the post-training adaptability of the model, we designed experiments to evaluate its plasticity under specific conditions. Specifically, we initialized the SOM-based global orientation detecting layer using the fully trained weights obtained from four orientations, and subsequently retrained the model using a reduced dataset that excluded orientations. As training progressed, the SOM gradually reorganized its structure. The model decreased the responses to the deleted orientations while the regions corresponding to the remaining orientations became more prominent. Moreover, the activation maps after retraining still overlapped significantly with those of the corresponding orientations, indicating that the model exhibits robust adaptability while maintaining its classification stability. Specifically, we reduced one specific orientation like Figure 7 position in an experiment. In this experiment, the reorganization pattern closely resembles the results shown in Figure 7, where only seven orientations were used from the beginning.

**Figure 9:**
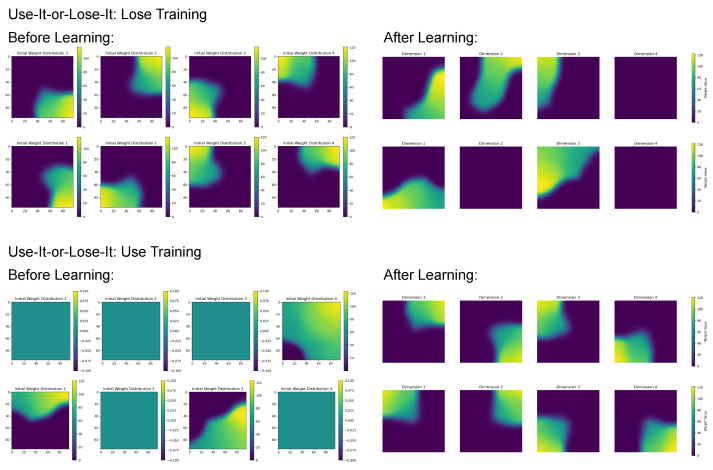
Use-It-or-Lose-It demonstration experiments result.

For demonstration, we used the saved SOM weights in the Section 3.6, which is about the experiments of single-orientation, double-orientation, and triple-orientation, and retrain them with the full-orientation dataset. The hyperparameters remained strictly invariant, whereas the initial network states were preseeded with the historically preserved weight matrices. For visualization, we chose some samples of the weight distribution of these experiments in Figure 9.

As shown in Figure 9, the retrained models exhibited significant reorganization of the SOM structure. In the models originally trained with limited orientations, previously inactive regions became responsive to the newly introduced orientations after full retraining. Conversely, models that initially possessed full orientation representation and then experienced orientation deprivation displayed a gradual reduction in the corresponding regions. This indicates that both sensory exposure and deprivation directly shape the functional topology of the orientation map. Whether through post-hoc recovery or induced forgetting, the SOM-based system consistently reflects the input statistics, confirming its capability to reorganize dynamically in response to environmental change. These findings reinforce the model’s biological plausibility, as they mirror use-dependent synaptic pruning and reinforcement observed in cortical development, show the strong biological theory-based interpretation of SOMAVS. This behavior reflects activity-dependent refinement processes observed in biological cortical circuits and provides further evidence that the classification mechanism of the visual system follows a use-it-or-lose-it principle, whereby functional representations are strengthened by experience and diminished in the absence of input.

### 3.8. Noise Immunity

To evaluate the robustness of SOMAVS, we conducted controlled experiments using two common types of artificial image corruption: Gaussian noise and salt-and-pepper noise. These tests were designed to simulate realistic visual disturbances while the model detects the orientation of a specific image. These noise modalities are mathematically standard yet highly representative of real-world physical sensor degradation: Gaussian noise effectively encapsulates thermal electronic noise and ambient dark-current fluctuations in complementary metal-oxide-semiconductor (CMOS) and charge-coupled device (CCD) sensors, while salt-and-pepper noise accurately replicates bit-transmission errors and pixel defects.

**Figure 10:**
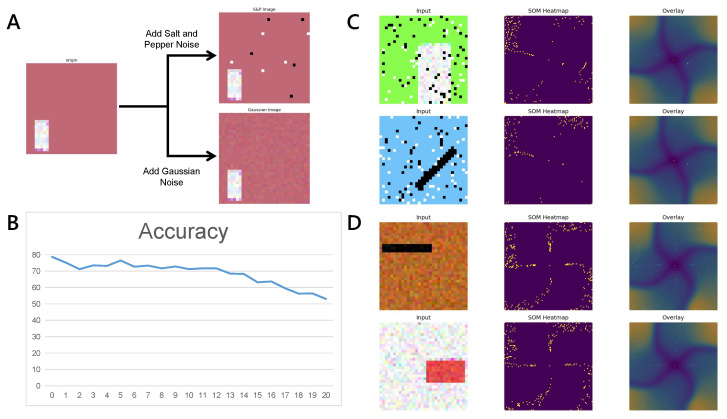
(**A**): Input examples with Gaussian and salt-and-pepper noise. (**B**): Accuracy under Gaussian noise with increasing σ. (**C**): Global activation under 10% salt-and-pepper noise. (**D**): Activation under σ=15 Gaussian noise.

For the Gaussian noise condition, we added zero-mean noise with a standard deviation σ ranging from 5 to 20. This range covers subtle to severe pixel-level disturbances, introducing increasing uncertainty in local orientation cues. For salt-and-pepper noise, we randomly altered a fixed percentage of image pixels to either minimum (0) or maximum (255) intensity values, with noise levels varying from 1% to 10% of the total pixels. This form of noise disrupts spatial continuity and introduces high-frequency corruption, particularly harmful for local filter-based systems. The visual examples of these two kinds of noisy inputs is shown in Figure 10A.

Despite these challenges, the SOMAVS model demonstrated strong resilience to both noise types. Table 4 reports the classification accuracy and NMI under each noise condition. As shown in the table, performance under salt-and-pepper noise degrades gradually, with accuracy remaining relatively high even at 10% corruption. This suggests that the AVS module’s LGN-like bio-inspired structure effectively mitigates the influence of sparse outliers such as separated noise pixels.

Under Gaussian noise, accuracy and NMI steadily decline as σ increases as shown in Figure 10B, the accuracy remains at a high level until the σ becomes much larger than the threshold of LGN structure. This suggests that SOMAVS is able to preserve global organization by averaging across noisy local activations.

For further illustration, the global activation maps under representative salt-and-pepper and Gaussian noise are shown in Figure 10C,D.

Even under substantial corruption (the possible influence in a realistic world), the activated regions largely correspond to the correct orientation classes. This indicates that SOMAVS encodes orientation using distributed and redundant population codes, similar to the noise-tolerant mechanisms observed in biological vision.

## 4. Discussion and Conclusions

In this study, we developed a biologically inspired Self-Organizing Map-based Artificial Visual System (SOM-AVS) that organizes orientation-selective representations in an unsupervised manner for static images. The experimental results demonstrated that the model effectively differentiates categories in a topologically organized manner, exhibiting clear functional segregation. Furthermore, it showed strong data efficiency, maintaining stable organization and discrimination performance even under limited sample conditions. These findings align well with biological visual system development, in which functional regions emerge in response to environmental stimuli, regardless of data abundance, reflecting the system’s plasticity and adaptability. In this study, the proposed SOM-AVS framework effectively demonstrates that topology-preserving, orientation-selective representations can emerge unsupervisedly from static images. Our empirical results confirm that the architecture achieves clear functional segregation and data efficiency, maintaining stable feature organization even under limited sample conditions. This adaptive emergence aligns with biological visual cortex development, in which functional tuning curves refine natively through passive environmental exposure rather than large-scale, supervised external feedback.

By simulating restricted perceptual experiences during training, the model displayed selective spatial organization patterns, which are consistent with patterns observed in the animal visual cortex. These results further validate our model’s biological relevance. Additionally, the plasticity experiments revealed that the system could dynamically reorganize its functional regions post-training, mirroring experience-dependent neural plasticity in biological systems. This supports the viability of SOMAVS as a computational framework for topological organization and adaptive structuring in bio-inspired visual processing.

Moreover, our analysis of the model’s robustness to noise revealed strong noise immunity across both Gaussian and salt-and-pepper conditions. When tested with varying levels of Gaussian noise and salt-and-pepper noise, the SOM-AVS model maintained stable performance, with only minor drops in organizational consistency and classification accuracy. This suggests that the SOM-AVS architecture inherently tolerates perturbations in local signals—likely due to its biologically motivated local-feature encoding and topology-preserving organization mechanism. Such resilience further enhances its suitability for real-world or biologically plausible visual processing.

However, several limitations remain in SOM-AVS. The current model primarily relies on simple two-dimensional static stimuli; future work should combine more complex natural scenes and dynamic video sequences to evaluate the scalability of topological organization under realistic visual statistics. While the SOM structure captures spatial topology well, scalability and efficiency in handling higher-dimensional and larger-scale data require further improvement. From a computational efficiency standpoint, the sequential winner-take-all metric computation required to establish the Best Matching Unit (BMU) across extensive iterations represents a non-trivial scalability bottleneck when adapting the system to complex engineering environments or scaling to massive pixel-level entry dimensions [39,40]. Moreover, the present model lacks hierarchical feedback mechanisms and multi-layered architectures common in biological visual pathways, which limits its ability to model higher-level organizational dynamics. It is important to note that our current SOM-AVS framework serves as a high-level computational abstraction of functional topology rather than a biophysically exact cortical model. Furthermore, while the exclusive reliance on synthetic stimuli adheres to strict developmental neurobiology paradigms for isolating variables, establishing the scalability of these mechanisms on unconstrained real-world natural images remains a critical challenge. To mitigate these systemic constraints and bridge our framework toward realistic motion-sensitive applications without inducing heavy computational overhead, our next-phase inquiry will focus on adapting the self-organizing layer to handle ultra-short time windows. Specifically, we aim to investigate how the network’s spatial topology reacts to transient, frame-to-frame spatiotemporal perturbations, thereby unlocking high-agility orientation and motion-direction co-selection within highly restricted temporal bounds [41].

In summary, the SOM-AVS model provides a biologically plausible, unsupervised learning approach to organize orientation-selective representations rather than explicitly predefining them, offering a topology-driven neural computational paradigm for understanding and constructing bio-inspired artificial visual systems. Future studies will focus on increasing model complexity and integrating neural plasticity mechanisms to enhance its biological reasonability and application potential.

In conclusion, the model demonstrated stable and robust performance across various training conditions, especially in scenarios with limited data and restricted perceptual experience. It effectively simulates developmental plasticity and experience-dependent topological organization observed in biological visual systems, providing computational insights into visual perception mechanisms in the cortex. Future work will aim to extend the model to handle more complex visual tasks and incorporate hierarchical feedback and multimodal integration, thereby improving its biological realism and practical applicability. The success of the SOM-AVS model highlights the promising potential of self-organizing neural networks as frameworks for unsupervised topological structuring of visual representations in bio-inspired vision.

## Figures and Tables

**Table 1:** Accuracy of SOMAVS on different datasets in basic training.

Dataset	Accuracy
Binary Images	95.47±2.34%
Full-color Images	78.71±2.87%

**Table 2:** Accuracy of SOM-AVS under different dataset modalities and data scales.

Dataset Configuration	Mean Accuracy ± Std. Dev. (%)
Binary Images (Full Data)	95.47±2.34%
Binary Images (50% Few-shot Data)	95.56±3.42%
Binary Images (10% Few-shot Data)	94.04±3.17%
Full-color Images (Full Data)	78.71±2.87%
Full-color Images (50% Few-shot Data)	76.02±5.82%
Full-color Images (10% Few-shot Data)	72.94±3.12%

**Table 3:** Comparison of model accuracy before and after integrating the SOM block in the ablation study.

Model	w/o SOM-Block	w/ SOM-Block	NMI
	Acc	Acc	
ResNet-style CNN	24.95±0.32%	33.16±0.81%	0.001
EfN-style CNN	28.53±0.51%	32.33±0.12%	0.008
ViT-hybrid-style CNN	24.28±3.38%	32.26±1.34%	0.012
AVS	74.12±1.88%	78.71±2.87%	0.610

**Table 4:** Accuracy in a noised scenario.

Model	Acc	NMI
1% Salt & Pepper Noise	80.31±3.47%	0.632
2% Salt & Pepper Noise	78.22±3.17%	0.600
5% Salt & Pepper Noise	73.01±4.83%	0.549
10% Salt & Pepper Noise	71.53±4.93%	0.481
Gaussian Noise σ=5	76.53±0.35%	0.537
Gaussian Noise σ=10	71.25±5.77%	0.464
Gaussian Noise σ=15	63.95±4.51%	0.281
Gaussian Noise σ=20	54.50±1.17%	0.151

## Data Availability

Our experimental data is available to reasonable request.

## Abbreviations

The following abbreviations are used in this manuscript:

LGN	Lateral geniculate nucleus
V1	Primary visual cortex
CNN	Convolutional neural network
ViTs	Vision Transformers
SOM	Self-organizing map
RGB	Red, green, and blue color channels
AVS	Artificial visual system
BMU	Best-matching unit
SNN	Spiking neural network
EfNB0	EfficientNet-B0
NMI	Normalized mutual information
CMOS	Complementary metal-oxide-semiconductor
CCD	Charge-coupled device
